# Signatures of adaptation and symbiosis in genomes and transcriptomes of *Symbiodinium*

**DOI:** 10.1038/s41598-017-15029-w

**Published:** 2017-11-03

**Authors:** Raúl A. González-Pech, Mark A. Ragan, Cheong Xin Chan

**Affiliations:** 1 Institute for Molecular Bioscience, The University of Queensland, Brisbane, QLD 4072 Australia; 2 School of Chemistry and Molecular Biosciences, The University of Queensland, Brisbane, QLD 4072 Australia

## Abstract

*Symbiodinium* is best-known as the photosynthetic symbiont of corals, but some clades are symbiotic in other organisms or include free-living forms. Identifying similarities and differences among these clades can help us understand their relationship with corals, and thereby inform on measures to manage coral reefs in a changing environment. Here, using sequences from 24 publicly available transcriptomes and genomes of *Symbiodinium*, we assessed 78,389 gene families in *Symbiodinium* clades and the immediate outgroup *Polarella glacialis*, and identified putative overrepresented functions in gene families that (1) distinguish *Symbiodinium* from other members of Order Suessiales, (2) are shared by all of the *Symbiodinium* clades for which we have data, and (3) based on available information, are specific to each clade. Our findings indicate that transmembrane transport, mechanisms of response to reactive oxygen species, and protection against UV radiation are functions enriched in all *Symbiodinium* clades but not in *P. glacialis*. Enrichment of these functions indicates the capability of *Symbiodinium* to establish and maintain symbiosis, and to respond and adapt to its environment. The observed differences in lineage-specific gene families imply extensive genetic divergence among clades. Our results provide a platform for future investigation of lineage- or clade-specific adaptation of *Symbiodinium* to their environment.

## Introduction

Dinoflagellates of genus *Symbiodinium* are known for their mutualistic relationships with corals and other marine organisms. Association with *Symbiodinium* enables corals to inhabit nutrient-poor tropical waters, grow and build up coral reefs; breakdown of the relationship leads to coral bleaching and, unless the relationship is re-established, death. Reef ecosystems in turn provide diverse benefits and services both to the environment, and to the economy of nearby communities^1^. A clear understanding of the relationship between *Symbiodinium* and corals is thus indispensable if we are to take a knowledge-driven approach to protect and manage these valuable ecosystems in the face of global environmental change.

*Symbiodinium* has been classified into nine distinct groups, clades A through I^2,3^. Studies based on genome and transcriptome data (generated so far only for *Symbiodinium* clades A through F) have contributed substantially to understanding the biology of each of these clades. One of those studies, based on transcriptome data, revealed that clades A and B use a smaller number of transcription factors than do other eukaryotes, implying particular gene regulation mechanisms^4^. Other studies report the genetic basis of thermal tolerance in clades C and D^5^, and gene homologs and pathways shared among clades A, B, C and D^6^. Another transcriptome-based study revealed that divergence of *Symbiodinium* within the same clade (clade B in this case) can be mirrored as extensive differences in gene expression^7^. To date, three draft genomes of *Symbiodinium* are available, revealing the presence of unique splice sites and a unidirectional gene arrangement in *S. minutum* (clade B)^8^, and that retrotransposition and gene duplication are the main drivers of gene family expansion in *S. kawagutii* (clade F)^9^. Comparative analysis of the *S. microadriaticum* (clade A) genome with the two others, together with additional sequence data from other dinoflagellates, supports the hypothesis that the symbiotic lifestyle of *Symbiodinium* was predisposed by an abundance of membrane transporters in all dinoflagellates, rather than being an adaptive novelty^10^.

Although some genome and transcriptome data are available from representatives of *Symbiodinium*, little is known of how gene content or biological function may differ within and between clades. Key questions about *Symbiodinium* biology remain largely unexplored, including what features distinguish them from other dinoflagellates, and what attributes are shared by all *Symbiodinium* or are exclusive to one or a few clades. To link genomic information to functions of cells, organisms and ecosystems, a comparative approach using gene families can be adopted^11^.

Here, using available genome^8–10^ and transcriptome^4,5,7,12–15^ data from dinoflagellates within Order Suessiales (*Symbiodinium* and *Polarella glacialis*) we systematically assess the gene families and inferred biological functions that are represented in one or more of *Symbiodinium* clades A through F, and investigate whether these functions are overrepresented in each analysed group. This represents the first comprehensive analysis of shared intra- and inter-cladal gene families in *Symbiodinium*.

## Results and Discussion

### Genome and transcriptome data

For this study we assembled 24 datasets (Table 1) of *Symbiodinium* genomes (3) and transcriptomes (19), and *P. glacialis* transcriptomes (2), with a total of 1,300,300 sequences (total length 1,333.87 Mbp; Supplementary Table S1). The N50 of each set of predicted coding sequences (CDS) ranges between 219 and 3987 bp (average 1480 bp). Fewer than 3% of the sequences from each dataset have significant BLASTn matches (*E* ≤ 10^−10^) against bacterial genomes, implying that there is little bacterial contamination. The completeness of each dataset was examined by comparison against core eukaryote genes in CEGMA^16^ and BUSCO^17^ (see Methods). On average, 72% of the 234 alveolate-stramenopile BUSCO genes^17^ and 89% of the 458 CEGMA genes^16^ were recovered by BLASTx from the datasets (Supplementary Table S1).

**Table 1:** Summary of the selected datasets for analysis in the present study.

Species (Isolates)	Clade	Number of sequences	N50 (bp)	Total length (Mbp)	Data type
*S. microadriaticum* (CCMP2467)^10^	A	49,109	3,987	166.722	Genome
*Symbiodinium* sp. (CassKB8)^4^	A	72,152	1,087	61.921	Transcriptome
*Symbiodinium* sp. (CCMP2430)^15^	A	44,733	1,356	42.483	Transcriptome
*Symbiodinium aenigmaticum* (mac04-487)^7^	B	45,343	1,355	44.628	Transcriptome
*Symbiodinium* sp. (SSB01)^12^	B	59,669	1,752	71.172	Transcriptome
*S. pseudominutum* (rt146)^7^	B	47,411	1,508	51.270	Transcriptome
*S. psygmophilum* (HIAp, Mf10.14b.02, PurPFlex, rt141)^7^	B	50,745	1,618	51.37	Transcriptome
*S. minutum* (Mac703, Mf1.05b, rt002, rt351)^7^	B1	51,199	1,597	57.248	Transcriptome
*S. minutum* (Mf1.05b)^8^	B1	47,014	2,675	97.202	Genome
*S. minutum* (Mf1.05b)^4^	B1	76,284	741	45.335	Transcriptome
*Symbiodinium* sp.^5^	C	26,986	534	12.546	Transcriptome
*Symbiodinium* sp.^54^	C	55,588	687	30.570	Transcriptome
*Symbiodinium* sp.^13^	C	65,838	1,746	97.581	Transcriptome
*Symbiodinium* sp.^15^	C1	45,782	1,443	45.706	Transcriptome
*Symbiodinium* sp. (MI-SCF055)^14^	C1	116,479	1,323	106.160	Transcriptome
*Symbiodinium* sp. (WSY)^14^	C1	131,066	1,239	113.375	Transcriptome
*Symbiodinium* sp.^15^	C15	37,277	1,299	33.008	Transcriptome
*Symbiodinium* sp.^5^	D	23,777	920	16.609	Transcriptome
*Symbiodinium* sp.^15^	D1a	43,662	804	25.956	Transcriptome
*Symbiodinium voratum* (CCMP421)^15^	E2	71,624	1,701	86.612	Transcriptome
*S. kawagutii* (CCMP2468)^9^	F	36,850	1,467	38.379	Genome
*S. kawagutii* (CCMP2468)^15^	F	11,679	219	2.666	Transcriptome
*Polarella glacialis* (CCMP1383)^15^	—	57,865	1,581	57.733	Transcriptome
*Polarella glacialis* (CCMP2088)^15^	—	32,168	1,161	21.755	Transcriptome

**Figure 1:**
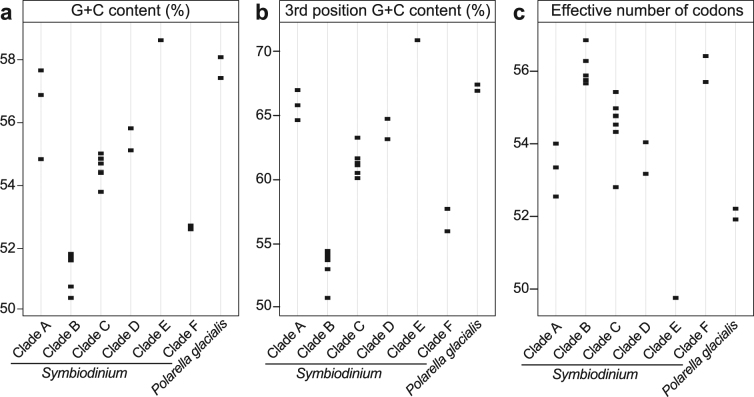
Overall G + C content (**a**), G + C content in third codon positions (**b**) and effective number of codons (**c**) are shown for the complete CDS of each dataset.

Where available, CDS predictions from the original source were used; otherwise CDS were predicted with TransDecoder v2.0.1 (see Methods). The overall sequence data yielded a total of 1,131,289 CDS with a total length of 1.21 Gbp; these correspond to the same number of predicted protein sequences. Completeness analyses returned results similar to those of the overall data (Supplementary Table S1). Overall G + C content ranges from 50.43% to 58.62% over all lineages (where *lineage* is defined as any clade of *Symbiodinium* or *P. glacialis*: Fig. 1a), G + C content in the third codon position between 50.81% and 70.86% (Fig. 1b), and the effective number of codons between 49.76 and 56.85 (Fig. 1c). These values differ between clades but fall within a relatively narrow range within each clade (Fig. 1).

Due to the heterogeneity and incomplete (and fragmented) nature of the transcriptome data, we carefully scope our analysis at the clade (instead of species or isolate) level. However, we note that most genes in dinoflagellates including *Symbiodinium* have been found to be constitutively expressed irrespective of growth conditions^18,19^. After pooling datasets by clade and removing redundant sequences (see Methods), our final datasets consist of 584,272 predicted proteins (Supplementary Tables S2 and S3). Details on the contribution of each individual dataset to the clade pools are shown in Supplementary Fig. S1.

### Delineation of gene families

Functions of proteins from each clade pool were assessed based on similarity search against the UniProt database, following Aranda *et al*.^10^ (see Methods). Of the 584,272 inferred proteins, 228,391 have significant (*E* ≤ 10^−10^) matches against sequences in UniProt (Swiss-Prot + TrEMBL); of these, 139,188 find a top match against an entry in Swiss-Prot, and a further 89,203 have a top match against an entry in TrEMBL. The matched UniProt identifiers were used to retrieve their associated KEGG Orthology (KO)^20^ and Gene Ontology (GO)^21^ terms. We define a *UniProt Homolog Group* (UP-HoG) as a set of proteins that share a common UniProt top match that has not been assigned a KO term, and a *KEGG Homolog Group* (KO-HoG) as those proteins for which the UniProt top match(es) have the same assigned KO term. We clustered proteins that show no significant (*E* ≤ 10^−10^) match against any UniProt entry using orthAgogue^22^ and MCL^23^, and define each of the resulting groups as an *orthAgogue-MCL Homolog Group* (OM-HoG).

The 228,391 proteins with UniProt matches were grouped into 40,688 UP-HoGs (mean size 3.85, sd 15.87) and 5679 KO-HoGs (mean size 15.39, sd 32.18), while those with no matches in the database were clustered into 37,483 OM-HoGs (mean size 3.47, sd 2.99); see Supplementary Table S4 for further details on size of the gene sets in each category. Because some dinoflagellate genes are similar in sequence to bacterial genes^24,25^, we carefully filtered these groups to minimize bacterial contamination (Supplementary Figure S2; see Methods) while attempting to retain true dinoflagellate genes. Most (83%) of the clusters with functional annotation (KO-HoGs and UP-HoGs) show no significant match against any bacterial sequence. Of those that do match a bacterial sequence, nearly one-third are eukaryote-like, with evidence of a multi-exonic CDS. We identified 4296 protein sets as having evidence of putative bacterial contamination, and excluded them from subsequent analysis. Of the 37,483 OM-HoGs, 36,318 (96.9%) have more than one representative in each lineage, and were retained for subsequent analyses. These steps yielded 78,389 protein sets (5331 KO-HoGs, 36,740 UP-HoGs and 36,318 OM-HoGs) for subsequent analysis. We provisionally refer to these sets as gene families.

### Functional annotation of gene families

For KO-HoGs and UP-HoGs, function was annotated at the protein level based on 62,339 distinct UniProt matches and their associated Gene Ontology terms (see Methods). For all gene families including OM-HoGs, we also searched for Pfam protein domains as additional support. In total, 33,766 of 78,389 (43%) families were annotated with 48,669 Pfam domains.

**Figure 2:**
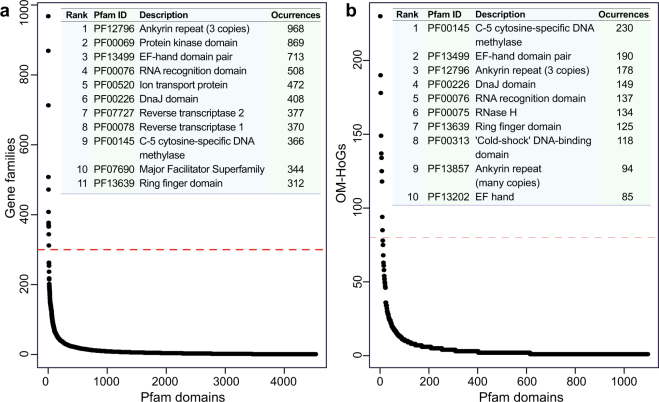
Number of gene families (*y*-axis) in which each Pfam domain (*x*-axis) was found in (**a**) all gene families, and (**b**) only OM-HoGs. The dashed red line separates the most-prevalent domains, >300 for all gene families and >80 in OM-HoGs, in each case. Identities of these domains are given in the top-right inset of each plot.

Figure 2a shows the ranked distribution of 4532 distinct Pfam domains found across all gene families, and the identity of those found in >300. *Ankyrin repeat (3 copies)* (PF12796), *protein kinase* (PF00069) and *EF-hand domain pair* (PF13499) domains were found in 968, 869 and 713 gene families. These functions are known to be prevalent in *Symbiodinium* ^8,12^. Ankyrins are important for protein-protein interaction (and potentially host-symbiont recognition), and the EF-hand domains are involved in calcium-binding and metabolism^8,26^. Membrane transport also appears prevalent in these gene families, *i.e.* *ion transport protein* (PF00520) and *major facilitator superfamily* (PF07690) in 472 and 344 respectively. The *DnaJ* domain found in 408 families are known to be involved in the response of *Symbiodinium* to photo- and thermal stress^14,27,28^. Similarly, *reverse transcriptase* (PF07727 and PF00078) domains in 370 and 377 families respectively may be involved in stress-response mechanisms^29–31^. The presence of *C-5 cytosine-specific DNA methylase* (PF00145) domains in 344 families agrees with the hypermethylated state of DNA in *Symbiodinium* ^32^ that might be also related to the regulation of gene expression in dinoflagellates^33^.

We used Pfam domains as a proxy of putative function of OM-HoGs. We recovered 1097 distinct domains distributed among 6283 OM-HoGs. Of these domains, ten were found in >80 families (Fig. 2b). These prevalent domains are largely similar to what we observed in the overall gene families (Fig. 2a). *RNase H* (PF00075) and *RNA recognition* (PF00076) domains, found in 134 and 137 families respectively, have been shown to regulate reverse transcription^34,35^ and splicing^36^. Two types of ankyrin repeat domains, PF12796 and PF13857, were found in 178 and 94 OM-HoGs respectively. As the functions implicated by these domains are critical to growth and survival, we included OM-HoGs in further analyses.

### Dynamics of gene families among *Symbiodinium* clades

**Figure 3:**
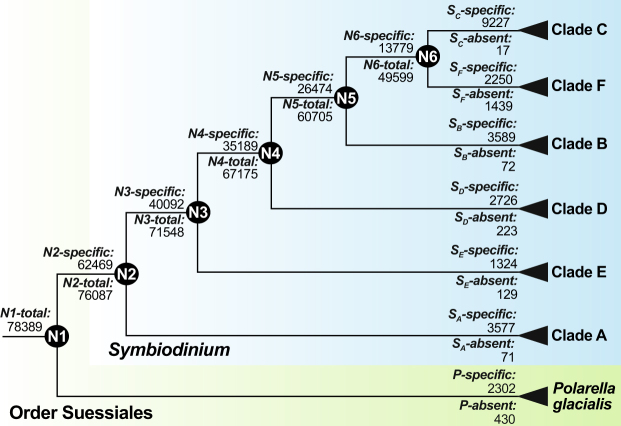
Changes in gene family numbers in Suessiales shown for *Symbiodinium* phylogeny (simplified cladogram based on Pochon *et al*.^37^), with *Polarella glacialis* as outgroup. The notation in this diagram is used throughout the text. Numbers of total and specific gene families at each node are shown to the left of the node in question. Numbers of specific and absent gene families for each lineage are correspondingly shown at the tips (right).

To explore the dynamics of gene families among *Symbiodinium* clades, we numbered each node (*N1* through *N6*: Fig. 3) on the accepted phylogeny^37^ and counted the families inferred to be represented at each node (Table 2). We infer a family to be part of *Node-total* at a node if a member of that family is identified in any lineage descendant from that node, regardless of whether or not the family is represented elsewhere in our dataset. *Node-specific* families are a subset of these, represented in one or more descendant clades but otherwise not observed in our dataset. The *N1-total* and *N1-specific* gene family sets are by this definition identical (Fig. 3); for simplicity we refer to these as *N1-total*. The membership of all gene sets is given in Supplementary Table S5.

**Table 2:** Number of gene families in which each lineage is found, shown for annotated (KO-HoGs or UP-HoGs) and non-annotated (OM-HoGs) gene families.

Lineage	Total	KO-HoGs/UP-HoGs	OM-HoGs
*Symbiodinium* clade A	30,409	15,399	15,010
*Symbiodinium* clade B	35,152	15,506	10,646
*Symbiodinium* clade C	43,412	23,121	20,291
*Symbiodinium* clade D	20,833	12,434	8,399
*Symbiodinium* clade E	17,481	10,910	6,571
*Symbiodinium* clade F	14,967	7,658	7,309
*Polarella glacialis*	15,920	9,195	6,725

The count of gene families differs substantially among clades (Table 2). These results may reflect actual genome dynamics (*e.g*. changes in genome size, gene content and/or sequence divergence) in the various lineages. However, for transcriptomes that lack genome data support, biases arising from the amount or quality of data (Supplementary Figure S3), taxon sampling, or details of data generation or processing (Supplementary Table S1) cannot be dismissed.

**Figure 4:**
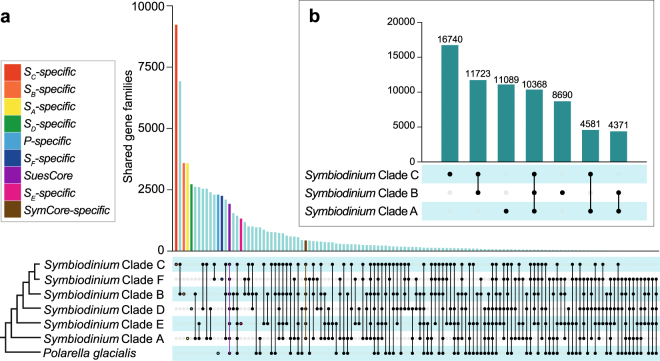
Gene families shared by (**a**) all individual lineages within Suessiales, and (**b**) clades A, B and C when compared among each other. The bars represent the number of gene families shared exclusively by the lineages marked below in the box with dots and connected by lines. In (**a**), lineage-specific gene families, those shared by all lineages in Suessiales (*SuesCore*) and *SymCore-specific* gene families are highlighted according to the colour code at the top right. The simplified topology shown at the bottom left depicts phylogenetic relationships among lineages.

To further explore the differences in gene family number among clades, we define lineage-specific gene families (*L-specific*, where *L* is an identified lineage, *e.g.* *L* = *S*_*A*_ denotes *Symbiodinium* clade A) as those represented in only that lineage, and *L-absent* families as those represented in all these lineages except *L*. The latter have either been lost from *L*, or are present but were not recovered in these data. Notation of gene families in individual lineages is given in Supplementary Table S6, and the number of shared gene families among all lineages is shown in Fig. 4a. The number of gene families specific to each lineage does not necessarily resemble the changes in gene family number displayed by the nodes. We assessed the effects of unbalanced taxon sampling and of differences in amount of data on the number of gene families inferred as specific to each clade (Supplementary Note and Supplementary Figure S4). Although we observed that taxon sampling could bias our results, the natural diversity of *Symbiodinium* could also contribute to the observed patterns. The amount of data, on the other hand, seems to impact our results less.

As transcriptome data are inherently incomplete, inferring the gain or loss of genes based on these potentially biased data is not straightforward. Here we discuss our results focusing on clades A, B (for which genome data are available) and C (the most data-rich lineage, with seven transcriptome datasets). *S*_*c*_ *-specific* gene families (9227) are the most abundant overall (Fig. 3), compared to 3577 *S*_*A*_ *-specific* and 3589 *S*_*B*_ *-specific* families. In contrast, *S*_*c*_ *-absent* (17) gene families are the least overall, compared to *S*_*A*_ *-absent* (71) and *S*_*B*_ *-absent* (72) families. The number of gene families specific to and absent from clades A and B are similar despite higher number of gene families in B (Table 2), suggesting no drastic gain of gene families between them.

In an independent analysis of shared gene families among clades A, B and C (Fig. 4b), the more-recently diverged C shares more gene families (11,723) with B than with the basal A (4581). Interestingly, *S*_*A*_ *-specific* families are more abundant than those shared by clade A with B, with C or with both, suggesting either a substantial gain of gene families in A, or an extensive loss of gene families between nodes N2 and N5 (Fig. 3). The latter alternative is supported by fewer families shared between clades A and B than between B and C. Under this scenario, our results suggest that clade C has retained more gene families and undergone further functional diversification than has clade B.

Similar gene-family dynamics are also observed in the numbers of *L-specific* and *L-absent* families in all other lineages (Fig. 3), although at this broad scale we cannot dismiss the impact of systematic and data biases such as poor taxa sampling in clades D, E and F (Supplementary Table S1 and Fig. S3), which could also contribute to this observation.

We categorized the gene families according to the lineages in which they are represented, defining those common to all *Symbiodinium* clades and *P. glacialis* as *SuesCore*, and those shared by all *Symbiodinium* clades (regardless of their presence or absence in *P. glacialis*) as *SymCore*. Families shared by all *Symbiodinium* clades but not *P. glacialis*, *i.e*. those exclusive to *Symbiodinium*, were annotated as *SymCore-specific*, and in this dataset are equivalent to *P-absent*. Given the possible combinations of gene-family sharing among these lineages (Fig. 4a), it is remarkable that families exclusive to one lineage are always amongst the major fractions, and more abundant than *SymCore-specific*. This could be explained by extensive divergence among lineages caused by differential recruitment (or preservation coupled with loss in other lineages), or alternatively by an extent of sequence variation so great as to prevent family members from clustering together with the strategies we employed. The low level of variation (sd ≤ 5 × 10^−11^) among *E* values, relative to the top Swiss-Prot match within each UP-HoG, renders the latter alternative less likely. Interestingly, the number of gene families shared by two lineages does not necessary correlate to their phylogenetic proximity. For instance, *Symbiodinium* clades C and F are closely related (Fig. 3, N6) but exclusively share fewer gene families (2302) than do clades C and B (6923), despite clade B diverging from the C-F lineage at a more-ancestral node (N5). However, clade F is the lineage with the fewest high-quality predicted proteins and the least-complete dataset (Supplementary Figure S1, Supplementary Table S2).

### What makes *SymbiodiniumSymbiodinium*?

For a functional overview at ordinal rank, we tested first for generality *versus* specificity by comparing *SuesCore* against all gene families in Suessiales (see Methods). GO terms enriched in *SuesCore* correspond to a wide variety of biological processes related to cytoplasmic translation, response to environmental factors (salt, temperature, nutrients, bacteria), and regulation of transcription and life cycle (Supplementary Table S7). Among the most-significantly overrepresented Pfam domains in *SuesCore* we found pentatricopeptide domains (PPRs), ankyrins, domains of AAA chaperone-like ATPases, several dynein domains and a kinesin motor domain (Supplementary Table S8); proteins carrying the latter three types of domain are necessary for movement and assembly of eukaryotic flagella^38,39^.

To determine what functions characterize *Symbiodinium*, and distinguish *Symbiodinium* from *Polarella* within Order Suessiales, we tested for enrichment of Pfam domains and GO terms in the *SymCore* and *SymCore-specific* gene families. GO terms and Pfam domains in *SymCore* were very similar to those enriched in *SuesCore*; this was expected, given that *P. glacialis* contributes few sequences to the latter gene family set (Supplementary Tables S9 and S10).

Amongst *SymCore-specific* gene families, enriched GO terms describe biological processes that are required for the maintenance of *Symbiodinium* symbiosis, including transmembrane transport of ions, amino acids and proteins^10^, mechanisms of response to reactive oxygen species (ROS), and protection against ultraviolet radiation (Supplementary Table S11). Reef habitats of *Symbiodinium* are typically characterised by high photon flux, and large amounts of ROS are generated during photosynthesis^40^. Mechanisms involved in nucleotide-excision DNA repair are overrepresented (and in *SuesCore* and *SymCore*), suggesting the critical involvement of this process in counteracting the mutagenic effects of UV radiation and free radicals in *Symbiodinium*. Enriched protein domains included those associated with transmembrane transport, protein-protein interaction potentially involved in host recognition (ankyrins and leucine-rich repeats)^41–43^, DNA repair, and protection from free radicals (Supplementary Table S12).

Since the enrichment tests compare general *versus* specific attributes, we expect lineage-specific functions to be underrepresented. For instance, multiple copies of genes encoding components of reverse transcription pathways have so far been reported only in *S. kawagutii* (clade F)^9^, and several domains annotated with that function are underrepresented in our *SuesCore*, *SymCore* and *SymCore-specific* gene families. Our results further suggest that certain protein domains considered as abundant in *Symbiodinium* may be dominant in specific genomes or clades; for instance, the domains involved in DNA methylation and transmembrane amino acid transport were underrepresented in *SuesCore* and *SymCore*, as is an EF-hand domain in *SymCore-specific* gene families.

### Lineage-specific enrichment of function

To assess lineage-specific attributes, we systematically identified gene families that are exclusive to, or absent in, each lineage (Supplementary Table S6). The G + C content distribution of the CDS in lineage-specific gene families resembles that of all gene families for that lineage (Supplementary Figure S5), suggesting non-exogenous origins (*i.e*. there is no evidence for systematic lateral gene transfer). GO terms and Pfam domains enriched in gene families exclusive to each lineage are not necessarily lineage-specific. For example, retrotransposition facilitated by reverse transcription has been reported in *Symbiodinium* clade F, a conclusion supported by our results (Supplementary Tables S13 and S14). However, gene families exclusive to clades A and B also display enriched GO terms and protein domains related to reverse transcription and retrotransposition (Supplementary Tables S15–18). Although viruses have been found in tight relationship with some *Symbiodinium* isolates in culture^44^, our results are not obviously the result of recent viral contamination since the G + C content of CDS associated with retrotransposition and reverse transcription does not differ from that of all CDS in Suessiales (Supplementary Figure S6). In addition, some retrotransposons are known to be activated under stress conditions in other eukaryotes including diatoms^29^ and plants^30,31^; our findings may reflect functions relevant to stress-response mechanisms in *Symbiodinium*. Other examples of GO terms and protein domains enriched in lineage-specific gene families that are not exclusive to a certain lineage include DNA methylation in *Symbiodinium* clades A and F (Supplementary Tables S15 and S19), and amino acid transmembrane transport in clades B and E (Supplementary Tables S18 and S20).

Among the biological processes annotated in clade A-specific gene families (*S*_*A*_ *-specific*), mechanisms related to adaptation to light conditions and avoidance of photodamage were enriched, including the GO terms *Chloroplast avoidance movement*, *Chloroplast localization*, *Establishment of plastid localization*, *Plastid localization*, *Chloroplast relocation* and *Phototropism*. Free-living isolates have been described in clade A^45,46^. These capabilities could be beneficial for free-living as well as symbiotic lifestyles, or for the ability to switch between the two. On the other hand, gene families absent only from clade A (*S*_*A*_ *-absent*) are rich in ribosomal protein domains and translational functions (Supplementary Tables S21 and S22).

Free-living isolates have been reported in *Symbiodinium* clade E as well, including the only isolate in this study. However, adaptive thermal regulation is the only biological process enriched in gene families exclusive to this clade that is obviously associated with the free-living habit (Supplementary Table S20). Many of the enriched functions are related to transmembrane transport, and the most-enriched protein domain in the exclusive gene families was the major facilitator superfamily (Supplementary Tables S20 and S23), a diverse family of membrane transporters implicated in the transport of metabolites and nutrients, including nitrate and nitrite^47^. Although membrane transport is a characteristic process of *Symbiodinium* symbioses and members of the major facilitator superfamily have been already reported for other *Symbiodinium* isolates^27^, this superfamily seems to have functions of particular relevance in this isolate from clade E.

Several of the most-enriched biological processes and protein domains in *S*_*C*_ *-specific* are linked to GTPase activity or its regulation, more specifically to the Rho GTPase family (Supplementary Tables S24 and S25). Rho GTPases function as molecular switches that activate responses to a wide variety of stimuli including changes in the cytoskeleton, regulation of gene expression, control of the cell cycle and transmembrane trafficking^48^. Rho-GTPase has been attributed to the rapid evolution of the Atlantic killifish *Fundulus heteroclitus* by facilitating adaptation to the presence of toxic compounds in the environment^49^. We therefore hypothesize that the overrepresentation of proteins with Rho GTPase-related functions, and the subsequent capability to respond effectively to different stimuli, could have contributed to the great genetic diversity observed in *Symbiodinium* clade C and its dominance in the Indo-Pacific ocean^50,51^.

*Symbiodinium* clade D are known for their high tolerance to thermal stress^52,53^. The molecular basis of this resilience has been linked to high proportions of unsaturated fatty acids in the cell membranes, protein folding, and chloroplast proteins involved in photosynthesis or constituents of the thylakoid membrane^5^. In this study we did not find any overrepresentation in *S*_*D*_ *-specific* of GO terms or Pfam domains annotated with plastid-related functions. However, the GO term *Unsaturated fatty acid elongation* is overrepresented in *S*_*D*_ *-specific* gene families. Among the overrepresented protein domains are a transcription factor DNA binding domain that regulates expression of heat shock proteins, and a heat shock protein (HSP20), both involved in protein folding in response to thermal stress (Supplementary Tables S26 and S27).

## Conclusions

The study of *Symbiodinium* from a genomic perspective, using both transcriptome and genome data, has broadened our understanding of its evolution, its capability to establish symbiosis and its response to a wide variety of conditions. Here we examined the gene families of six *Symbiodinium* clades (A-F) to identify functional attributes either shared among, or exclusive to, each of them. We also used data from the closely related species *Polarella glacialis* to determine which features are characteristic of *Symbiodinium* within the order Suessiales. Gene families shared among all these *Symbiodinium* are enriched in functions essential to the establishment and maintenance of symbiosis, and survival in a high-energy environment. At the same time, clade-specific differences in the presence or absence of gene families, and in the enrichment of functions, offer potential for members of distinct clades to specialize in diverse environments. Our results provide a foundation for future investigation of lineage- or clade-specific adaptation of *Symbiodinium* to their environment, and emphasize the need for more high-quality genomic data from understudied *Symbiodinium* clades and closely related species (such as *Polarella glacialis*).

## Methods

### Data collection and preparation

We collected a total of 30 datasets (Supplementary Figure S1), from which 24 were selected for this study (Table 1) based on quality of assembled sequences and certainty of taxonomic assignment (Supplementary Figure S7), including the published genomes of *S. minutum* (clade B)^8^, *S. kawagutii* (clade F)^9^ and *S. microadriaticum* (clade A)^10^, and 21 transcriptomes (19 from *Symbiodinium* spp. and two from *Polarella glacialis*) from previous studies^4,5,7,12–14,54^ and from the Marine Microbial Eukaryote Transcriptome Sequencing Projects database (MMETSP)^15^. Characteristics of the datasets are summarised in Supplementary Table S1. Because different methods yield different estimates of completeness^55^, we compared each dataset with the 458 CEGMA genes^16^ (BLASTx, *E* ≤ 10^−10^) and the BUSCO^17^ datasets for eukaryotes, alveolates-stramenopiles, and protists (using BUSCO v3.0.2b and by BLASTx, *E* ≤ 10^−10^). Sequences in each dataset were additionally searched (BLASTn, *E* ≤ 10^−10^) against all bacterial genomes in RefSeq release 76 to assess the proportion of sequences from bacterial sources (putative contaminants).

Where available (*i.e*. for the three genomes and the transcriptomes from MMETSP), the predicted CDS and proteins were used for the analyses. For the other transcriptome data, we used TransDecoder v2.0.1 (transdecoder.github.io) to predict CDS and proteins at default settings. Completeness of the protein datasets was assessed with CEGMA^16^ and BUSCO^17^ genes, as for the original data but using BLASTp instead of BLASTx. Detail for each CDS/protein dataset is shown in Supplementary Table S1. Codon usage of full-length CDS (*i.e*. CDS that begin with a start codon and end with a stop codon) in each dataset was assessed using *chips* and *cusp* from the EMBOSS software suite (emboss.sourceforge.net). Proteins from *Symbiodinium* isolates within the same clade were pooled together: three datasets in clade A, seven in B, seven in C, two in D, one in E and two in clade F. Similarly, all proteins from the two *P. glacialis* isolates were pooled as one. Redundant sequences from each clade pool were removed using CD-HIT^56^ to cluster similar sequences at default settings (sequence identity threshold = 0.90); the longest sequence in each group was kept as representative.

### Homolog clusters

To assess protein functions we followed Aranda *et al*.^10^ using BLASTp search (*E* ≤ 10^−10^) against the UniProt database (release 2016_01). Briefly, protein sequences were first searched against Swiss-Prot, and those with no matches were subsequently searched against the TrEMBL database. The UniProt identifier of the best match for each protein was used to retrieve its associated KEGG Orthology (KO)^20^ term using UniProtKB ID mapping release 2015_03 (ftp.uniprot.org/pub/databases/uniprot/current_release/knowledgebase/idmapping) and the Gene Ontology (GO)^21^ terms in UniProt-GOA release 163 (ftp.ebi.ac.uk/pub/databases/GO/goa). Proteins without functional annotation were clustered using orthAgogue^22^ v1.0.3 (e-value cut-off = 10^−10^) and MCL^23^ (*I* = 1.2, scheme 7), as recommended for extensive genetic divergence (expected among *Symbiodinium* clades^1^); here we define a group of two or more such proteins as an OM-HoG.

To minimize the inclusion of sequences from potential bacterial sources (*i.e*. contaminants) in our analysis, we carefully selected a high-confidence set of putative homolog groups of *Symbiodinium* and *Polarella glacialis* for subsequent analysis based on the schema detailed in Supplementary Fig. S2. All KO-HoGs and UP-HoGs in which no member matched a bacterial sequence were included in subsequent analysis. For groups in which one or more members matched a bacterial sequence, we referred to the genome data of the corresponding isolate where available. We took the presence of multiple exons in these CDS as evidence of eukaryote origin; homolog groups containing any protein with such evidence were retained for subsequent analyses. For homolog groups in which one or more members matched a bacterial sequence but no genome data were available, and those for which some members had bacterial hits but no multi-exon evidence, we kept any groups in which the bacterial hits are present in two or more lineages; these are potential real dinoflagellate proteins that may have arisen through lateral genetic transfer from a bacterial source^24,25^. Among the non-annotated OM-HoGs, we considered only those containing proteins from two or more of the original 24 datasets.

### Functional analysis of gene families

For our purposes here, we refer to the selected homolog clusters as *gene families*. We based our functional annotation on the Gene Ontology (GO) terms. Pfam domains^57^ were annotated for the proteins corresponding to each gene family using PfamScan^58^ (*E* ≤ 0.001). For each category in Supplementary Table S6, GO and Pfam-domain enrichment analyses were performed against *N1-total* as the reference background; here we consider a gene family as the unit of analysis. GO enrichment analysis was performed using the topGO Bioconductor package^59^ implemented in R v3.2.1, applying Fisher’s Exact test with the ‘elimination’ method to correct for the dependence structure among GO terms. A one-tailed Fisher’s Exact test was used to assess over- and under-representation of Pfam protein domains independently, with adjustment of *p*-values for multiple tests following Benjamini and Hochberg^60^.

### Data availability

Datasets analysed during the current study are identified and cited in this published article (and its Supplementary Information files).

## Electronic supplementary material

Supplementary Information

Supplementary Tables
