# Supplementary material for: Signatures of adaptation and symbiosis in genomes and transcriptomes of Symbiodinium
Source: Sci Rep. 2017 Nov 3;7:15021. doi: 10.1038/s41598-017-15029-w (PMC5670126; doi:10.1038/s41598-017-15029-w)
Supplement: Supplementary file 1 — Supplementary Information [file 41598_2017_15029_MOESM1_ESM.pdf]

Supplementary Information

Signatures of adaptation and symbiosis in genomes and transcriptomes of *Symbiodinium*

Raúl A. González-Pech[1], Mark A. Ragan[1] and Cheong Xin Chan[1,2]*

[1]Institute for Molecular Bioscience, The University of Queensland, Brisbane, QLD 4072, Australia

[2]School of Chemistry and Molecular Biosciences, The University of Queensland, Brisbane, QLD 4072, Australia

*Corresponding author (c.chan1@uq.edu.au)

Supplementary Note

Impact of taxon sampling and data amount on gene-family analysis

Unbalanced taxon sampling is a universal issue in comparative microbial genomics and transcriptomics. Some taxa are intrinsically species-poor (or poorly known), others are species-rich (or well-sampled), and genomes are often of different sizes and complexity. More is usually gained by learning from the world as we find it than by excluding real data to fit an idealised model.

Among the datasets used in this study, clades C, B, A, D, F, and E are represented by 7, 4, 2, 2, 1, and 1 species. To assess the impact of taxon sampling on our gene-family analysis, we systematically assessed the number of lineage-specific families across the 78,389 gene families by rarefying the clade B and C datasets to two species at a time. With seven species in B and four in C, there are a total 126 (${}^{7}C_{2} \times {}^{4}C_{2}$) possible combinations, and for each combination the number of species represented by clades C, B, A, D, F, E, and *Polarella glacialis* are 2, 2, 2, 2, 1, 1 and 1. The results for these 126 assessments are shown in Supplementary Figure S4; the number observed based on the non-rarefied data for each lineage is denoted by a red asterisk.

In general, the number of lineage-specific families based on the rarefied data is higher than for the non-rarefied data, except for clades B and C. The data points from clade B (and probably A) form two distinct clouds, while those in C range across a single broad distribution (Supplementary Figure S4A). This result points to biases in phylogenetic coverage and/or diversity, especially for the hyperdiverse clade C. Therefore rarefying the data, while statistically sensible, does not adequately capture the diversity of *Symbiodinium* either within or among clades.

To further assess biases related to the amount of data (i.e. number of proteins) from each clade we rarefied clade representation in the overall families. Here we used clade E (found in 17,481 families; Table 2) as the lower-bound reference, and clade A (found in 30,409 families; Table 2) as the upper-bound reference for rarefication. We denote Z as the maximum number of gene families to contain a specific clade, and set Z = 18,000 (lower bound), 24,000, and 30,000 (upper bound). For instance, at Z = 18,000 we first removed for each clade (of A through F), members from any gene families at random until there were no more than 18,000 families that contain representatives of that clade, before assessing the number of lineage-specific families; we did this in 500 replicates. The data points for these 500 replicates are shown in Supplementary Figures S4B (Z = 18,000), S4C (Z = 24,000) and S4D (Z = 30,000); the results based on non-rarefied data are shown in red asterisks.

In contrast to what we observed in Supplementary Figure S4A, the number of families specific to each lineage (i.e. each case of Supplementary Figures S4B, S4C and S4D) is largely consistent, with little variation. The numbers based on rarefied data are higher than in the non-rarefied data, except for clade C. As expected, the numbers based on rarefied data are more similar to those based on non-rarefied data as Z is increased from 18,000 to 30,000. Interestingly, for clades A and B at Z = 24,000 these numbers converged. These results strongly imply that our results are less sensitive to the biases in amount of data than to species selection in each lineage.

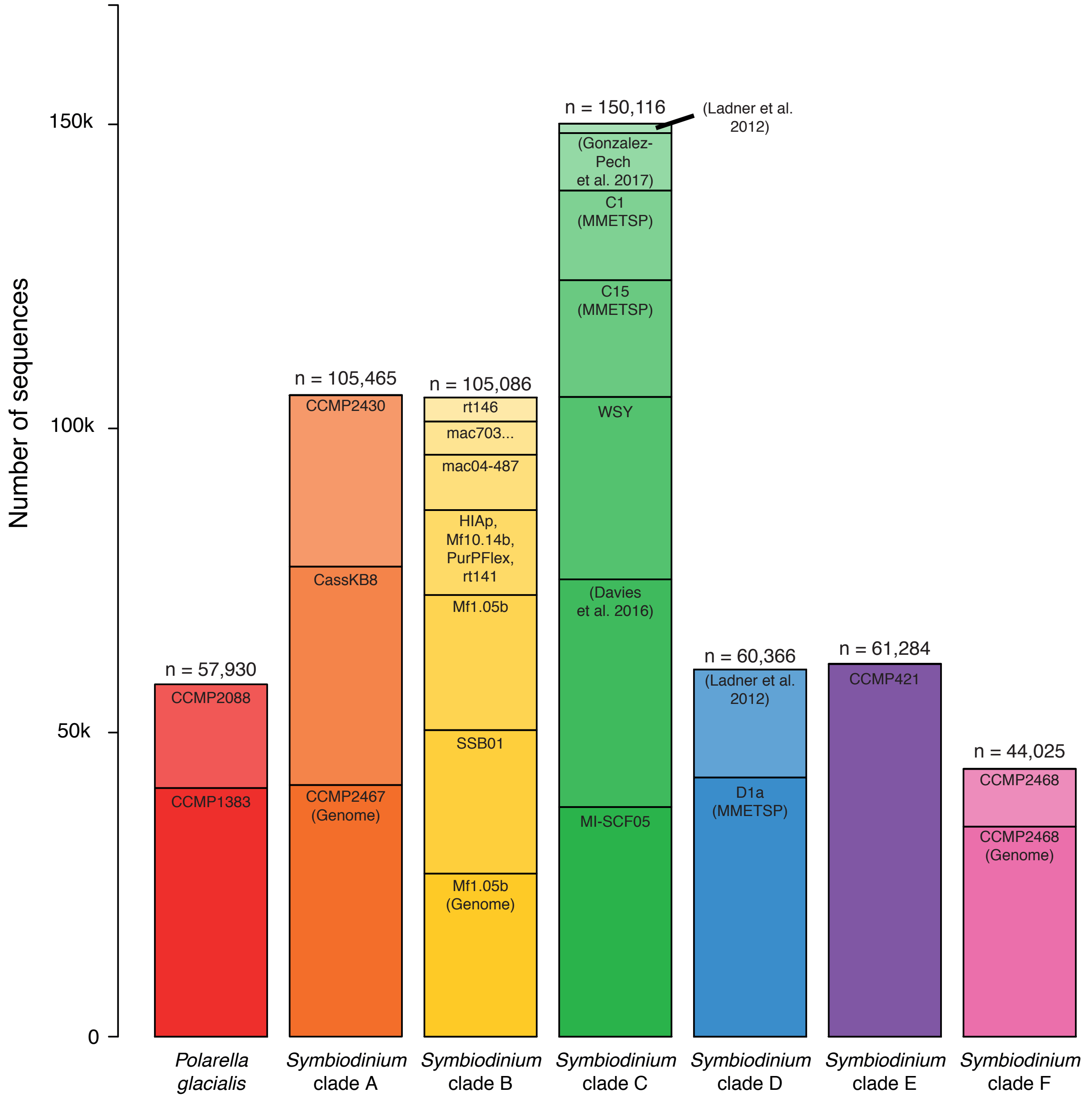

Supplementary Figure S1. Contribution of individual datasets to the clade data pools. Each *Symbiodinium* clade (as well as *P. glacialis*) is represented with a different color and individual datasets at each clade pool is shown in a different shade. Datasets with the largest contribution to the clade pool are at the base of each bar with decreasing contribution towards the top. Individual datasets IDs is based on the strain names and, if missing, on the reference literature. Genomic datasets are specified with a note between brackets and occur at the base of their respective clade bar, indicative of their full-length proteins. The total number of proteins in each clade pool is displayed at the top of the bars.

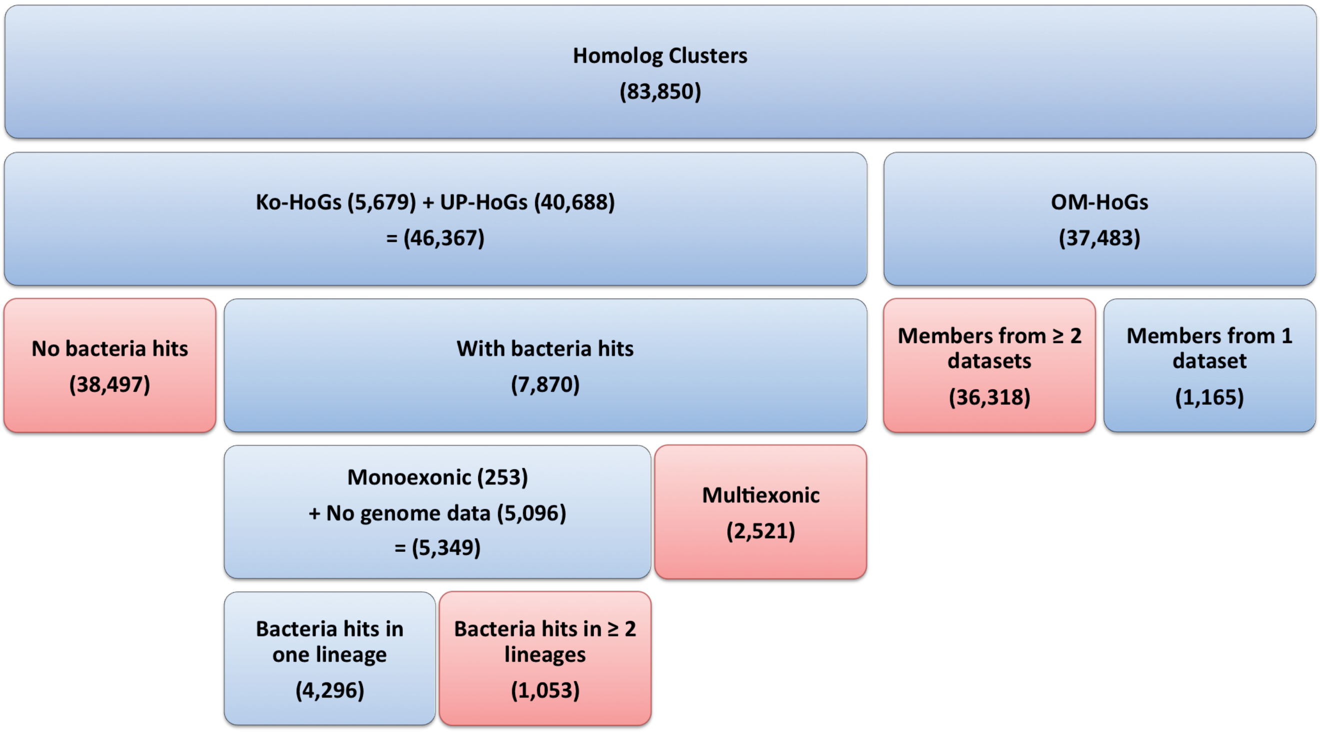

Supplementary Figure S2. Breakdown of the selection criteria for the homolog clusters found in *Symbiodinium* and *P. glacialis*. The number in parenthesis indicates the number of clusters that fell into each category. Red boxes represent the clusters selected as gene families.

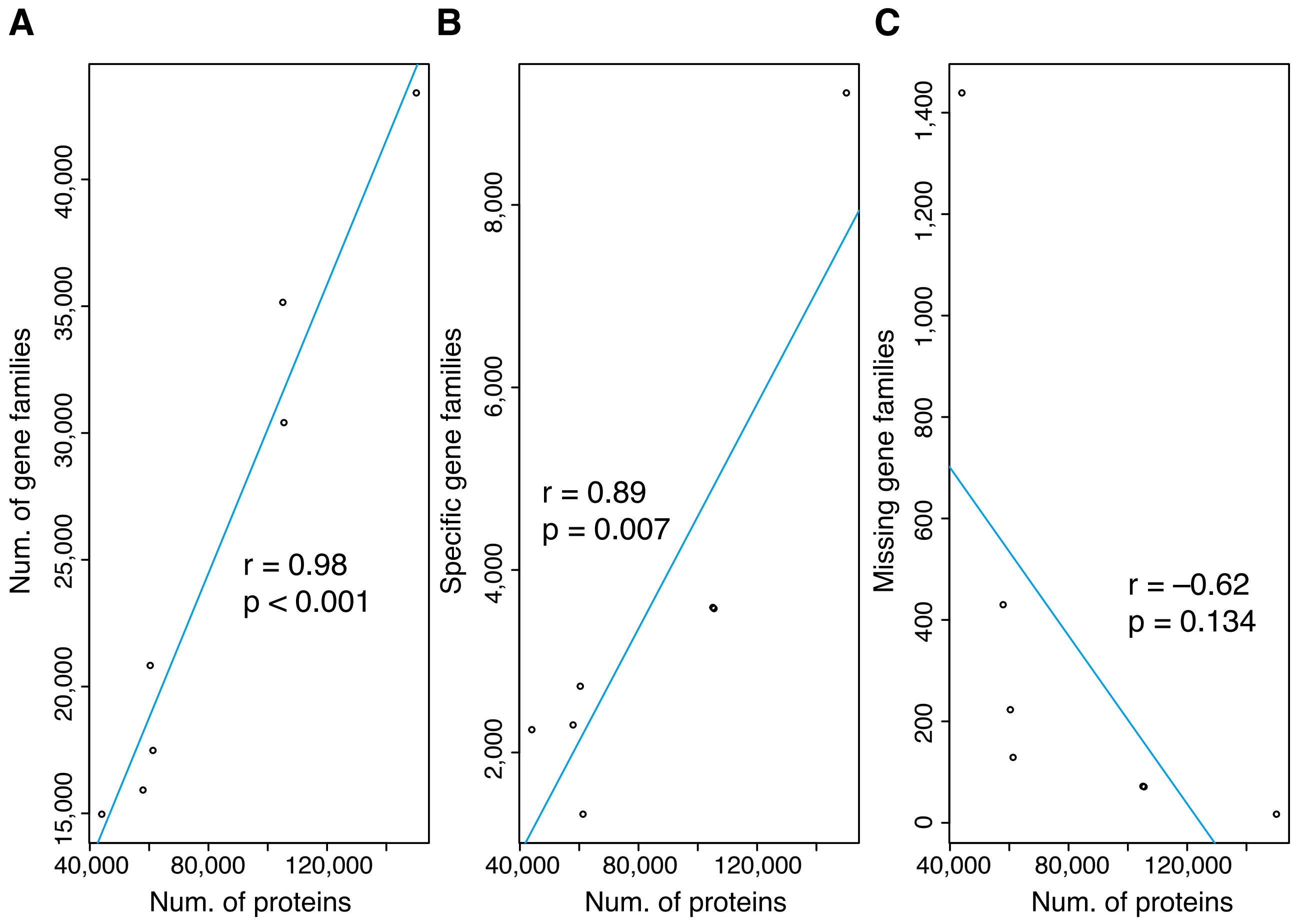

Supplementary Figure S3. Correlation of the number of proteins used as input with the total **(A)**, specific **(B)** and missing **(C)** number of gene families for each lineage.

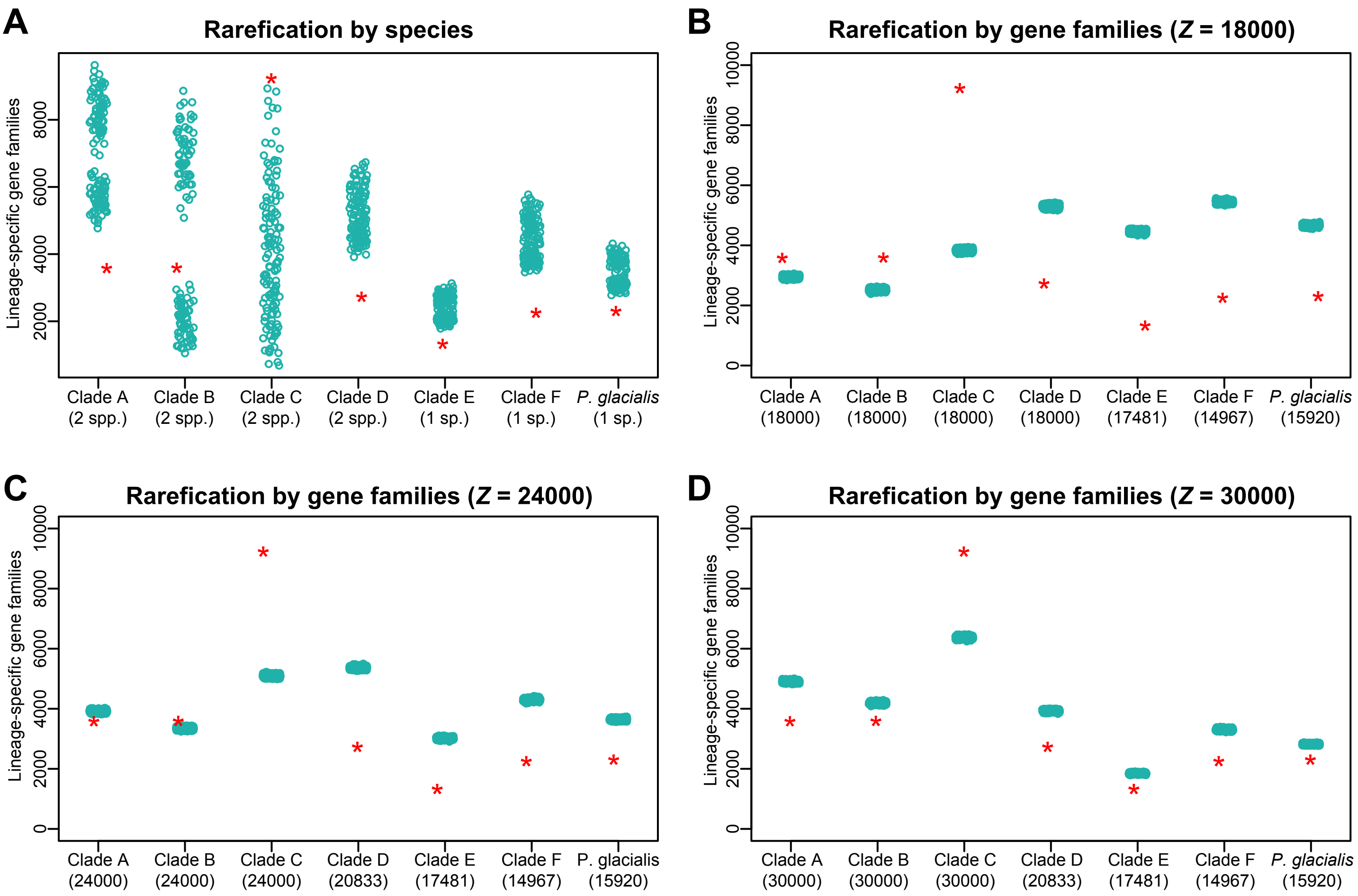

Supplementary Figure S4. Rarefication analyses for number of species per lineage and number of gene families. The red asterisks show the number of gene families specific to each lineage based on non-rarefied datasets, and the turquoise circles the results based on the rarefied data. In the species rarefications **(A)**, each point corresponds to one of 126 possible combinations with the shown number of species per lineage. Rarefication of gene families was done at three different thresholds *Z* = 18000 **(B)**, 24000 **(C)** and 30000 **(D)**, and each point represents one of the 500 replicates; the number of gene families after rarefication is shown in brackets for each lineage.

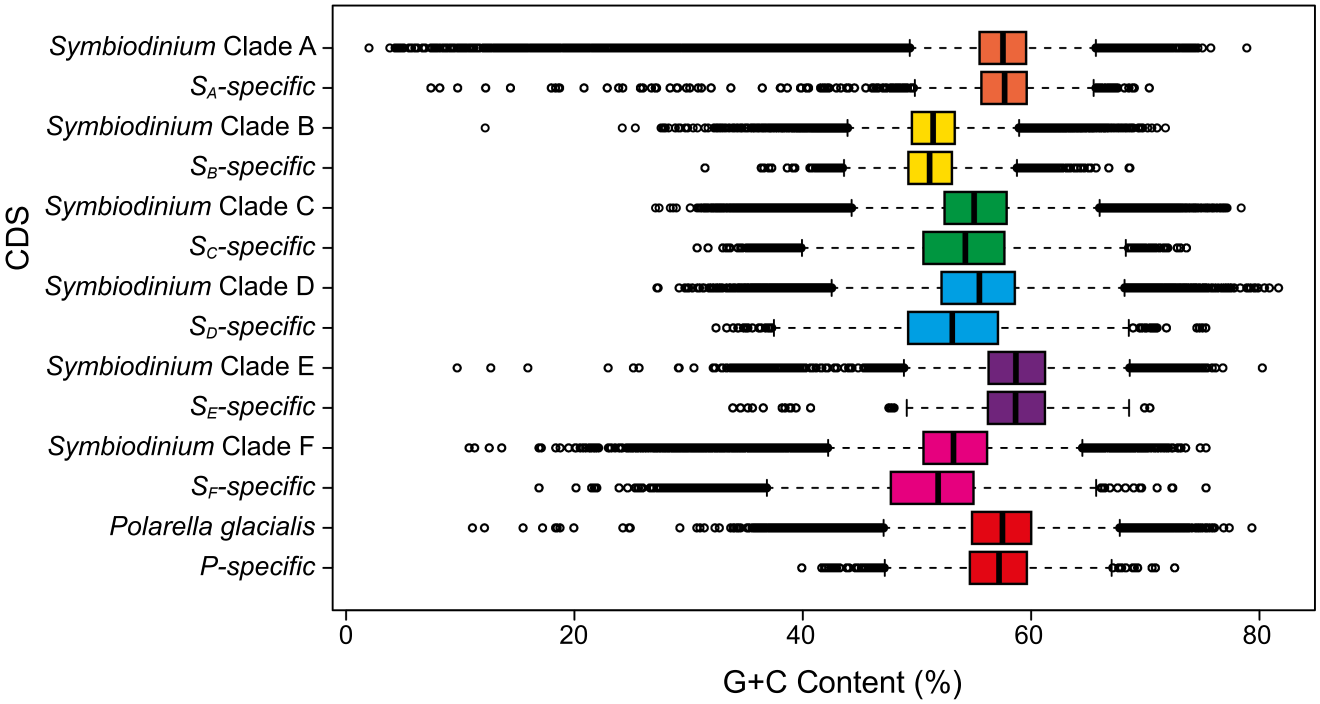

Supplementary Figure S5 G+C content in Suessiales CDS of lineage-specific gene families (following notation in main text) compared to the CDS of all gene families for the corresponding lineage. Different colours represent different lineages.

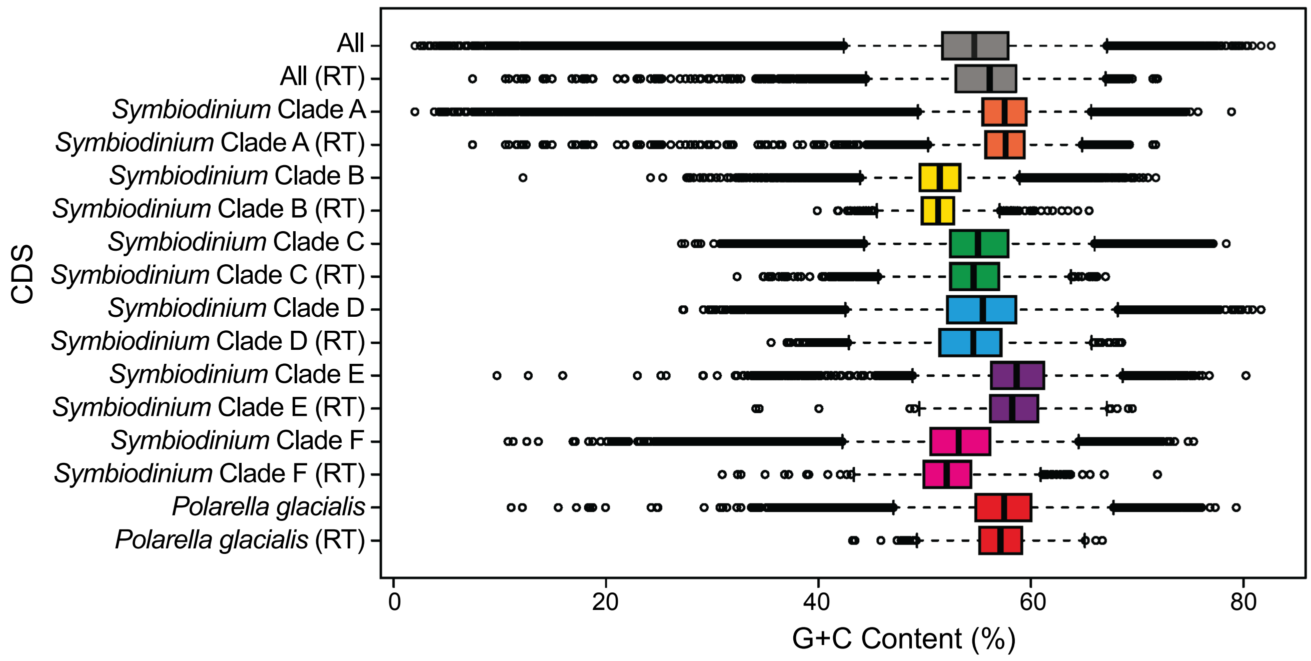

Supplementary Figure S6. G+C content in Suessiales CDS with functions involved in retrotransposition and reverse transcription compared to all CDS. Different colours reprsent different lineages and colour grey represents all lineages combined. CDS with retrotransposition functions are noted as RT.

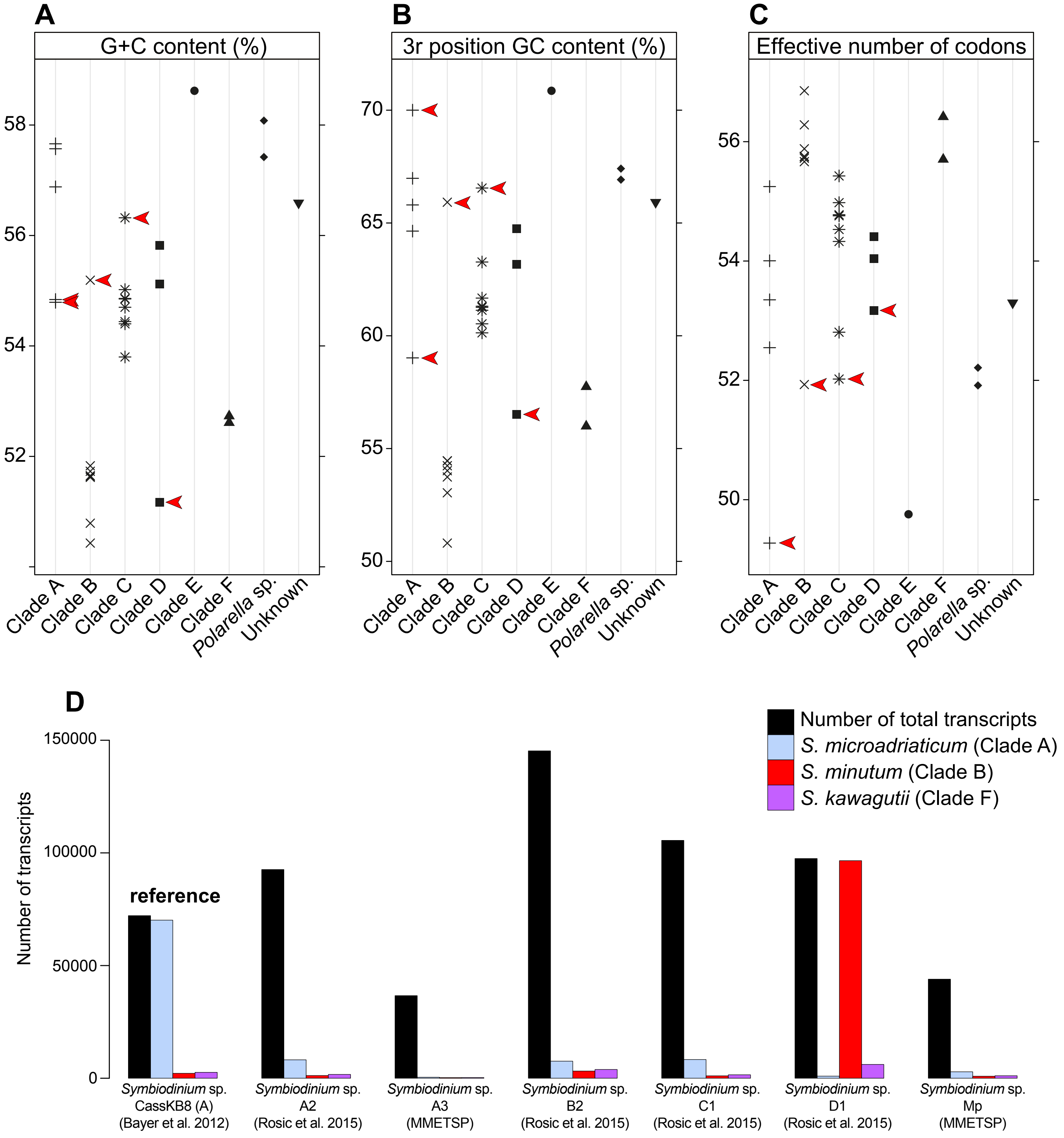

Supplementary Figure S7. (A) Overall G+C content, **(B)** G+C content in third codon positions and **(C)** effective number of codons usage for the complete CDS of each dataset. Each distinct shape of the data point represents the corresponding lineage. The outlier datasets from each lineage marked with a red arrowhead were removed from this study. **(D)** Verficication of clade identity for the outlier datasets with ambiguous clade assignment, based on mapping of transcripts onto published genomes of *S. microadriticum* (Clade A), *S. minutum* (Clade B) and *S. kawagutii* (Clade F). As a reference for each dataset, the number of total transcripts is shown (black bar). The mapping of CassKB8 transcriptome (Clade A) is included as a reference.
